# Usefulness of matrix metalloproteinase-7 in saliva as a diagnostic biomarker for laryngopharyngeal reflux disease

**DOI:** 10.1038/s41598-021-96554-7

**Published:** 2021-08-23

**Authors:** Nu-Ri Im, Byoungjae Kim, Kwang-Yoon Jung, Seung-Kuk Baek

**Affiliations:** 1 Department of Otorhinolaryngology-Head and Neck Surgery, College of Medicine, Korea University Anam Hospital, 73, Inchon-ro, Seongbuk-gu, Seoul, South Korea; 2 College of Medicine, Neuroscience Research Institute, Korea University, Seoul, Republic of Korea

**Keywords:** Biomarkers, Diseases, Medical research

## Abstract

Several diagnostic methods are currently being used to diagnose LPRD (laryngopharyngeal reflux disease), but have the disadvantage of being invasive, subjective, or expensive. Our purpose in this study was to investigate the correlation between pepsin and MMP-7 (Matrix Metalloproteinase-7) in pharyngeal secretions of subjects according to RSI (Reflux Symptom Index) score to find out the diagnostic value of MMP-7. We recruited 173 subjects aged between 19 and 85 years who completed the RSI scale. All samples were taken after waking up, and the amount of the pepsin and MMP-7 in saliva were measured by means of an enzyme activity assay. There was a significant increase of pepsin and MMP-7 activity in the study group with an RSI score of 13 or higher. The sensitivity and specificity of MMP-7 for predicting the possibility of an RSI of 13 or more was higher than that of pepsin. When MMP-7 and pepsin were combined, this sensitivity and specificity increased. An enzyme assay of MMP-7 in saliva may be a noninvasive and easy technique for diagnosing LPRD.

## Introduction

Laryngopharyngeal reflux disease (LPRD) is elicited by back flow of food, stomach acid, and pepsin from inside the stomach through the esophagus to the throat^1^. LPRD is one of the most common and important disorders of upper airway inflammation disease^2^. The common symptoms of LPRD include throat clearing (98%), persistent cough (97%), globus pharyngeus (95%), and hoarseness (95%)^3^.

Currently, LPRD is diagnosed by using a questionnaire on symptoms, laryngoscope examination, monitoring of *p*H in the pharynx and esophagus, and response to empirical treatment. First of all, LPRD is diagnosed using a questionnaire, the Reflux Symptom Index (RSI). If the RSI score is 13 or higher, it can be diagnosed as LPRD, which results have been reported to be reproducible and valid^4^.

Laryngoscopy examination is widely used for diagnosis, but it can be subjective, because nonspecific signs may be provoked by laryngeal irritation and inflammation, and the results may differ depending on the examiner^5^. A 24-h *p*H monitoring test is suggested as a gold standard diagnosis for LPRD. It records pharyngeal and esophageal *p*H changes for 24 h, which allows physicians to make an objective diagnosis for LPRD^6^. However, this monitoring test is invasive and costly, and cannot be performed on all patients^7–9^. Therefore, in the actual clinical field, if there are symptoms or signs that are suspected to be from LPRD, empirical treatment to diagnose LPRD is done by checking whether the symptoms or signs are improved after LPRD medication with a proton pump inhibitor (PPI). The empirical treatment is to use the PPI two or three times a day and see if there is recovery from symptoms^10,11^. Furthermore, just as there is no objective diagnostic method readily available to outpatients, there is no clear and effective treatment for LPRD. Thus, treatment for LPRD ranges from dietary and lifestyle modifications, such as weight loss, smoking cessation, abstaining from alcohol, and no sleep right after meals, to medications such as PPI and prokinetics^12–15^. Although the most well-known and standard drug is PPI, there has not yet been enough evidence to conclude that PPI treatment is superior to a placebo in random trials^16,17^. Patients with LPRD often do not show good symptomatic improvement with these treatments^18^.

Recently, a diagnostic method using pepsin, which is known as a cause of the disease, was developed. This method measures the amount of pepsin contained in saliva using a pepsin activity assay. Previous studies have suggested that pepsin concentration in saliva may be significantly correlated with gastroesophageal reflux disease (GERD); so it provides a noninvasive method using saliva^8,19–21^.

Our previous study suggested that Matrix Metalloproteinase-7 (MMP-7) plays a role in the degradation of E-cadherin in human pharyngeal epithelial cells exposed to acid. This phenomenon has similarly been investigated in histological findings in patients with LPRD^22^. Therefore, our purpose in this study was to compare the RSI score of patients who were not diagnosed with LPRD with the concentrations of pepsin and MMP-7 in their pharyngeal secretions, in order to investigate the correlation and to propose a noninvasive method for diagnosing LPRD.

## Materials and methods

### Subjects

**Figure 1:**
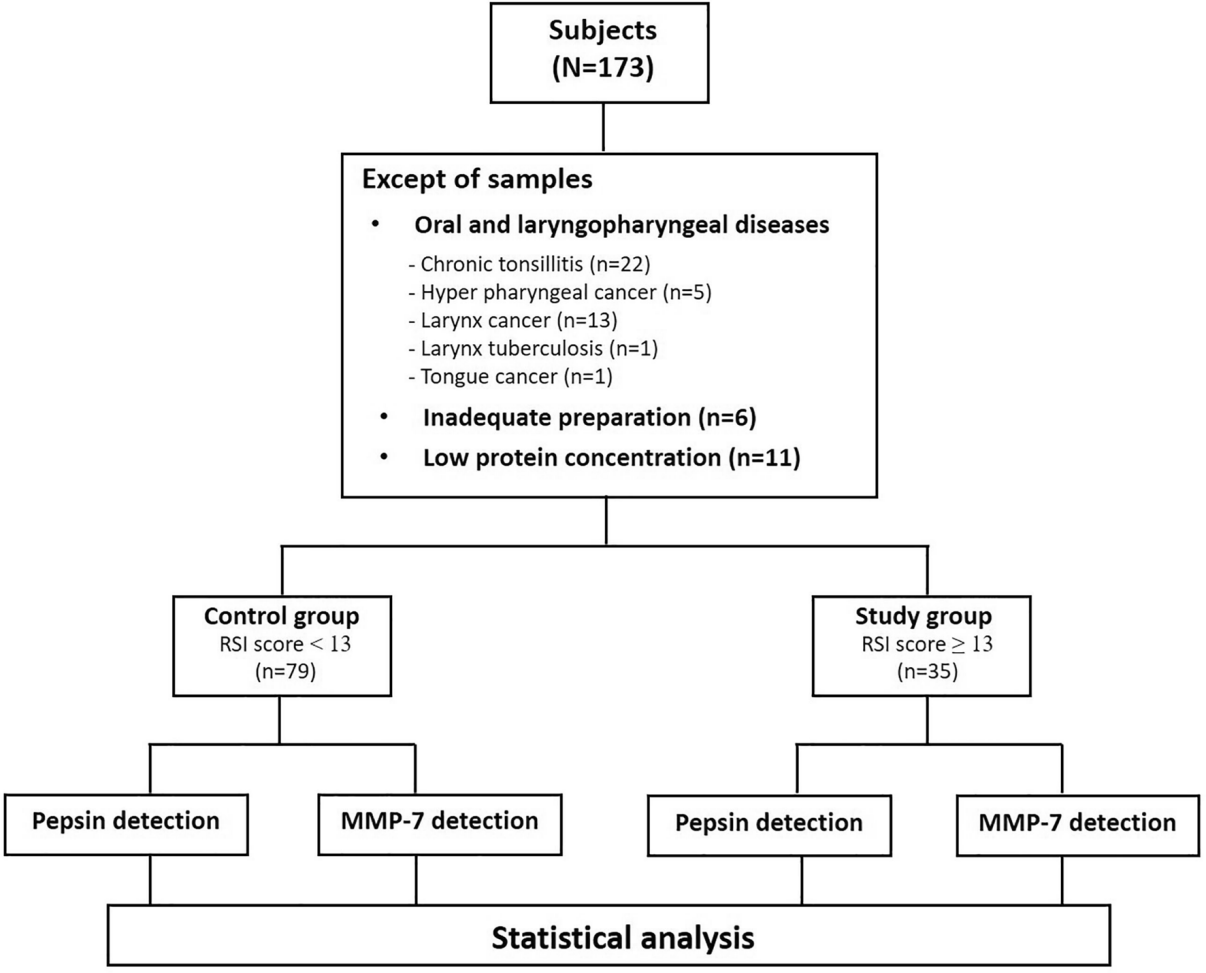
Flow chart of the study. RSI = Reflux symptom index, MMP = Matrix Metalloproteinase.

Among 173 healthy volunteers, we excluded 59 who had oral and laryngopharyngeal diseases, such as acute/chronic infectious or inflammatory diseases and cancers, inadequate preparation of saliva, or low protein concentration within samples (Fig. 1). Thus, we selected 114 healthy volunteers (79 men and 35 women; age range, 19–85 years) who had not been diagnosed with LPRD for our sample population. We harvested saliva from the pharyngeal area in the oral cavity of all subjects, who completed the RSI questionnaire. A score below 13 points was considered to be the control group, and subjects with an RSI of 13 or higher were considered to be the study group. Protocols and experimental design parameters were reviewed and approved by the Institutional Review Board of Korea University Hospital. We obtained informed consent from all participants, and carried out all methods in accordance with the relevant guidelines and regulations.

### Salivary collection and protein concentration measurement

Saliva was collected before breakfast. Eating, drinking, gargling, and tooth brushing were forbidden before saliva collection. To collect saliva and mucosal secretion within the pharynx, the sample solution was gathered in a conical tube after gargling with 10 ml of distilled water. Then, centrifugation was performed to remove solids, and the supernatant was used for measurement. We transferred the samples to a refrigerator at 4 °C and analyzed then within two days. We estimated the amount of protein in each saliva sample according to the Bicinchoninic Acid (BCA) protein assay, using a standard concentration curve of bovine serum albumin.

### Western blot

For the Western blot analysis, we mixed 10 ug of protein from each sample with 5 × Laemmli buffer and 5% *β*-mercaptoethanol and boiled it for 10 min. Briefly, we separated an equal volume of protein by SDS–polyacrylamide gel electrophoresis and transferred it to a nitrocellulose membrane. We then incubated the membranes overnight at 4 °C with a 1:1000 dilution of E-cadherin (clone number H-108, catalog number sc-7870; Santa Cruz Biotechnology, Inc, Dallas, Tx, USA), MMP-7 (catalog number AV46075; Sigma-Aldrich, St. Louis, MO, USA), or a 1:2000 dilution of β-actin antibody (clone number C4, catalog number sc-47778, Santa Cruz Biotechnology) for loading control in a blocking solution. Next, we incubated the membranes with a 1:1000 dilution of the appropriate anti-rabbit (catalog number sc-2004; Santa Cruz Biotechnology) or anti-mouse antibody (catalog number sc-2005; Santa Cruz Biotechnology) in the blocking solution. We viewed blots using the chemiluminescence kit (Santa Cruz Biotechnology), and captured the images with ChemiDoc (Bio-Rad Laboratories, Hercules, CA, USA).

### MMP-7 enzyme substrate assay

For the activity measurement of MMP-7, using the 5-ug protein salivary sample, we incubated the concentrate with 10 μM APMA (4-aminophenylmercuric acetate) in assay buffer for 1 h at 37 °C. After the addition of dilute MMP-7 substrate (5-FAM/QXL™ 520 FRET peptide) (SensoLyte 520 MMP-7 assay kit; Anaspec, Inc., Fremont, CA, USA) 1:100 in assay buffer and incubation for 1 h at 37 °C, we read the samples at an excitation wavelength of 490 nm and emission of 520 nm (SOFTMAX PRO v5 software, Molecular Devices, Sunnyvale, CA, USA) with a SpectraMax M2^e^ plate reader (Molecular Devices).

### Pepsin measurement

To quantify the pepsin activity in the saliva samples, using the pepsin/pepsinogen assay kit (Abcam, Cambridge, UK), we incubated the concentrate of 1:10 pepsin substrate solution A in assay buffer for 10 min at 37 °C, then read the samples at an excitation wavelength of 328 nm and emission of 418 nm (SOFTMAX PRO v5 software, Molecular Devices) with a SpectraMax M2^e^ plate reader (Molecular Devices).

### Statistics

We used the SPSS version 20.0 statistical software (IBM SPSS, Armonk, NY, USA) and Prism Version5.0, GraphPad, for data processing. Each experiment was repeated 3 times to obtain statistical significance. We used logistic regression to evaluate the correlation between pepsin activity and MMP-7 activity. In addition, in order to obtain the predicted cut-off value of LPRD diagnosis, we did receiver operating characteristic (ROC) curve analysis using salivary pepsin concentration and salivary MMP-7 concentration, and obtained sensitivity and specificity values accordingly. Statistical significance was considered to be *p* < 0.05.

## Results

### Reflux symptom index score

Among the 114 subjects, 79 had an RSI score below 13 points (control group, 54 men and 25 women; age range, 19–81 years) and 35 had an RSI score of 13 or higher (study group, 23 men and 12 women; age range, 19–85 years) (Fig. 1).

### Optimal time of saliva collection

To identify the optimal time when an adequate concentration of protein can be collected from the sample, we did a preliminary experiment with 10 subjects (5 men and 5 women; age range, 19–37 years). Subjects collected samples 8 times a day for 5 days: after waking up, before or after breakfast, before or after lunch, before or after dinner, and before bedtime (Supplementary Figure S1A).

We measured soluble E-cadherin (extracellular segment of E-cadherin) and MMP-7, which were significantly associated with the pathogenesis of LPRD in our previous study^22^. We found that the protein expression levels of MMP-7 and soluble E-cadherin increased most from waking up until before breakfast (Supplementary Figure S1B). As well, MMP-7 enzyme activity was highest during the same period (Supplementary Figure S1C). Thus, all samples were collected after waking up and before breakfast.

### Comparison of pepsin and MMP-7 enzyme activity in saliva samples according to RSI score

**Figure 2:**
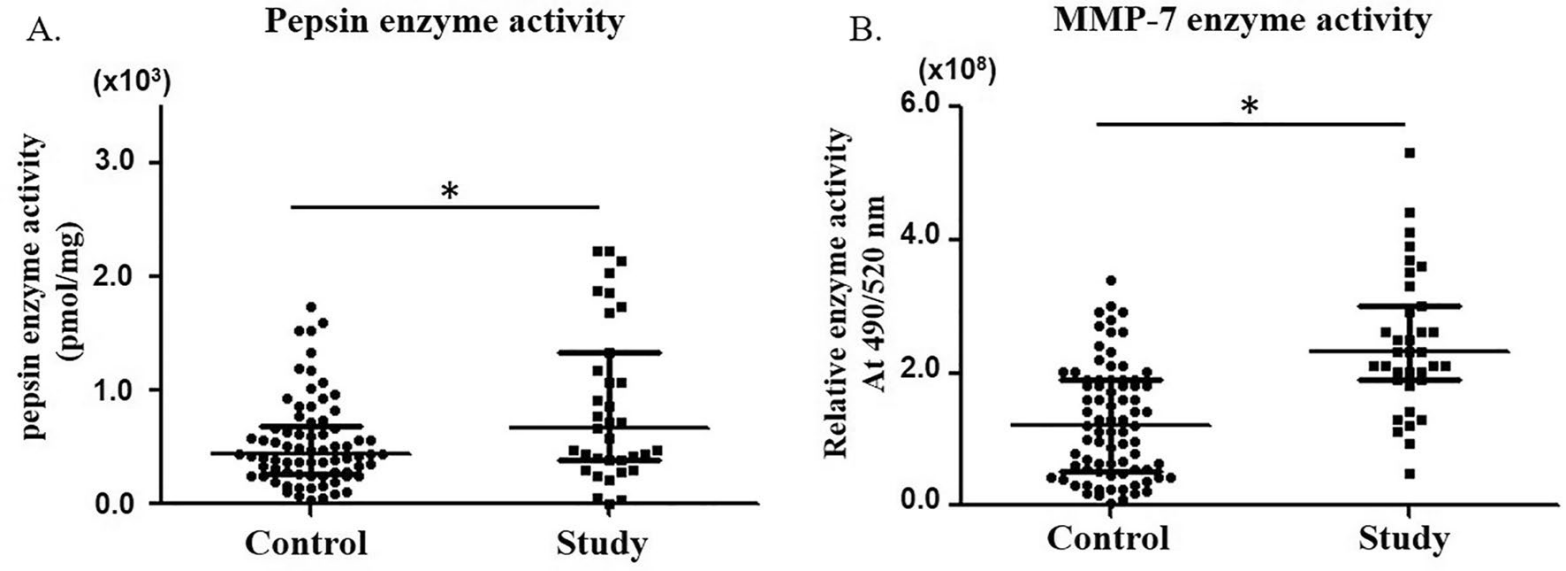
Enzyme activities of pepsin and MMP-7 in control and study groups. (**A**) Pepsin. (**B**) MMP-7. Horizontal bars represent median value (central horizontal bar) and 25th and 75th percentile values (lower and upper bars) for each group.

The median enzyme activity of salivary pepsin and salivary MMP-7 of the control and study groups were 0.440 × 10^3^ (range, 0.265 × 10^3^ to 0.666 × 10^3^) and 0.666 × 10^3^ (range, 0.381 × 10^3^ to 1.252 × 10^3^) pmol/mg; 1.2 × 10^8^ (range, 0.53 × 10^8^ to 1.9 × 10^8^) and 2.3 × 10^8^ (range, 1.9 × 10^8^ to 2.95 × 10^8^) RFU/min, respectively, which were statistically significantly different between the two groups (*p* < 0.05) (Fig. 2A). As well, the median activity of salivary MMP-7 of the control and study groups was 1.2 × 10^8^ (range, 0.53 × 10^8^ to 1.9 × 10^8^) and 2.3 × 10^8^ (range, 1.9 × 10^8^ to 2.95 × 10^8^) RFU/min, respectively, which was statistically significantly different between the two groups (*p* < 0.05) (Fig. 2B). The significant increase of the activity of pepsin and MMP-7 in the study group with an RSI of 13 or higher means that their saliva contained more pepsin and MMP-7. Thus, it may be possible to infer that the study group is associated with LPRD.

### Diagnostic value of pepsin and MMP-7 for LPRD

**Figure 3:**
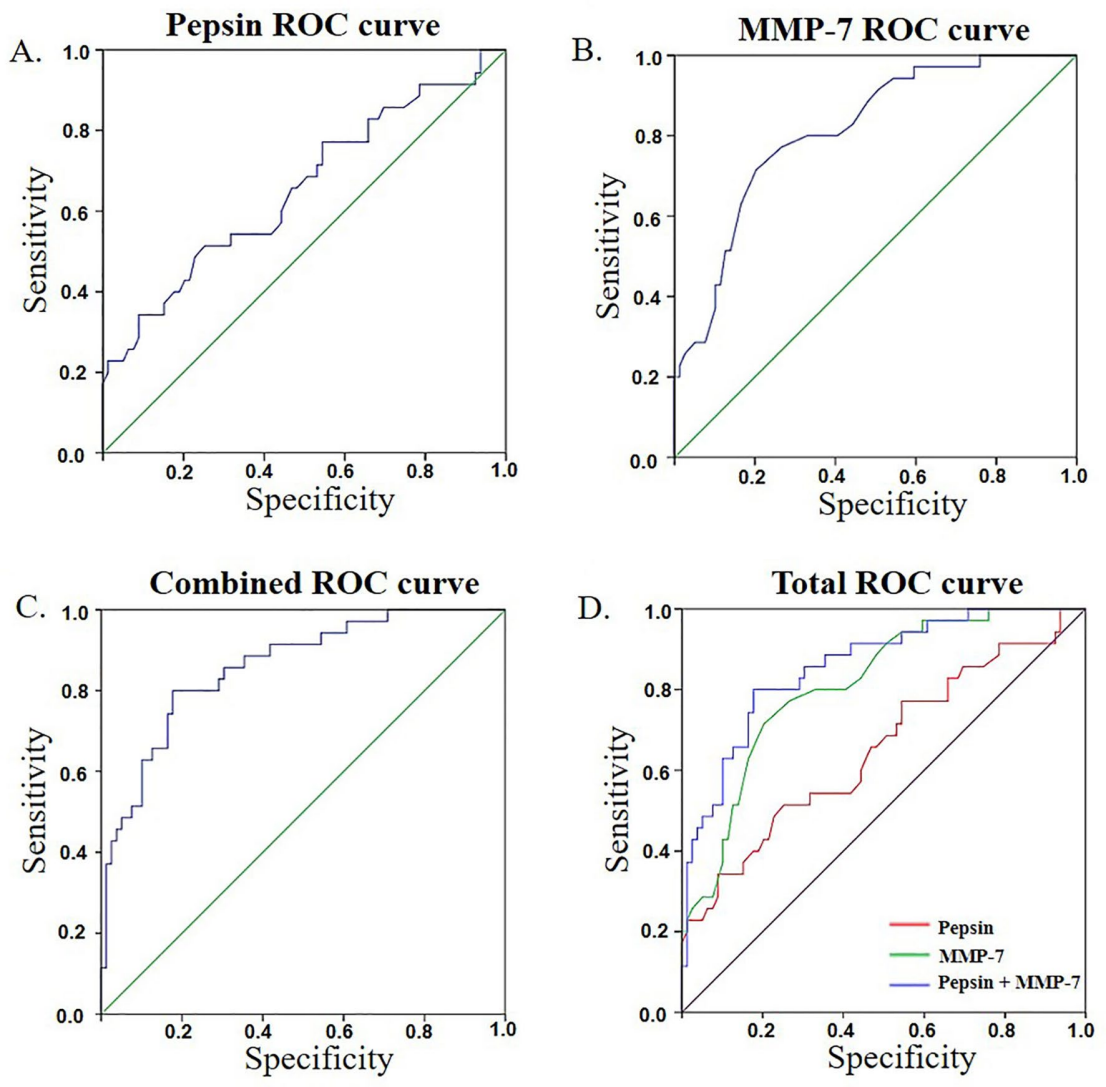
Receiver operating characteristics curve analysis for finding an optimal cut-off value of salivary pepsin and MMP 7 concentration needed to identify LPRD patients. (**A**) ROC curves generated with pepsin (blue line), base (green line) (**B**) ROC curves generated with MMP-7 (blue line), base (green line) (**C**) ROC curves generated with combined pepsin and MMP-7 (blue line), base (green line) (**D**) ROC curve total combining (**A**) (yellow line), (**B**) (green line) and (**C**) (blue line) and base (purple line).

In the ROC curve analysis to identify the optimal cut-off value of salivary pepsin and MMP-7 according to an RSI score of 13, the area under the curve (AUC) of pepsin was 0.654 (sensitivity 51.43, specificity 74.68, *p* < 0.05) and that of MMP-7 was 0.813 (sensitivity 71.43, specificity 79.75, *p* < 0.05) (Figs. 3A and 3B, Table 1). In addition, the AUC of a combination of pepsin and MMP-7 was 0.859 (sensitivity 80, specificity 82.8, *p* < 0.05) (Fig. 3C, Table 1). Therefore, in the total ROC curve, the diagnostic value was highest in a combination of pepsin and MMP-7, followed by MMP-7 (Fig. 3D, Table 1).

**Table 1:** Statistical significance of pepsin and MMP-7 according to RSI score.

Group	AUC	Criterion	Sensitivity	95% CI	Specificity	95% Cl	*p*-value
Pepsin	0.654	> 0.64 × 10^3^	51.43	34.0–68.6	74.68	63.6–83.8	0.048
MMP-7	0.813	> 1.9 × 10^8^	71.43	53.7–85.4	79.75	69.2–88.0	0.042
Combine	0.859	> 0.333	80.00	63.1–91.6	82.28	72.1–90.0	0.037

AUC, area under the curve; CI, confidence interval.

These results show that the disease prediction is higher than that of conventional pepsin when using MMP-7 alone, and that the diagnosis prediction increases when MMP-7 is combined with pepsin.

## Discussion

The main diagnostic method for LPRD that is most commonly practiced clinically is empirical medication with a proton pump inhibitor, although there are other diagnostic methods, such as laryngoscopy examination, 24-h double *p*H monitoring, and manometry^15^. Thus, although many studies are currently under way to find an objective and noninvasive diagnostic strategy for LPRD, there is still no definitive diagnostic method^23^.

Recently, the diagnostic value of pepsin has emerged as a noninvasive, low-cost salivary pepsin detection method^8,19–21,24^. Pepsin is a specific biomarker that can detect gastric reflux in saliva, because it is produced only in the stomach^19^. After the first use of the salivary pepsin assay to analyze saliva/sputum pepsin in GERD using the fibrinogen digestion method was published, Western blotting and enzyme-linked immunosorbent assay (ELISA) methods were used to detect pepsin^25,26^. Furthermore, the rapid lateral flow test was developed to provide rapid analysis and predictive values^26^. However, since pepsin can be detected in LPRD as well as in GERD or GERD-related disorders, it might show low sensitivity and specificity values. Therefore, in order to develop a more specific diagnostic tool for LPRD, we focused on the diagnostic feasibility of MMP-7, which was associated with E-cadherin degradation, part of the pathogenesis of LPRD, in our previous study^22^.

Belafsky et al. developed an index using nine questions to document the severity of LPRD symptoms^4^. The RSI data were derived from personal databases without evidence of LPR, and results were compared before and after PPI prescriptions. The mean pretreatment RSI of LPR patients was significantly higher than that of the control group, and after 6 months of PPI treatment, the mean RSI of LPR patients was close to that of the asymptomatic control group. The indicator is considered abnormal if the RSI score is 13 or higher. In our present study, when we divided a population into a study group and a control group based on a RSI score of 13, the activities of pepsin and MMP-7 showed a significant difference between the two groups.

In the previous studies of an association between RSI score and pepsin in LPRD patients, higher pepsin levels in sputum were associated with higher RSI and RFS scores^27,28^. However, considering that pepsin is activated at an acidic condition below *p*H 4, pepsin levels as well as the presence of acid reflux should be evaluated to diagnose LPRD. Acid reflux can be assessed by 24-h double *p*H monitoring, but this diagnostic technique is expensive and invasive, and thus is not used in a real clinical setting. Our previous study demonstrated that the MMP-7 plays an important role in acidic damage to the pharyngeal mucosa in an LPRD in vitro model made by *p*H 4 acid exposure. Therefore, in this study we proved the correlation with RSI using two main causes, pepsin and acid-induced MMP-7, which cause damage when LPRD occurs.

In addition, to measure the prediction of disease diagnosis, we evaluated clinical sensitivity and specificity using the ROC curve based on the activity of pepsin and MMP-7 in the normal and study groups. When only the activity of pepsin was used as a diagnostic marker, the sensitivity and specificity were 51.43% and 74.68%, and results were similar to those of a previous study^29^, in which the presence of pepsin in the saliva of patients with chronic cough and vocal-cord dysfunction induced during LPR were found in 87% of patients with an RSI score of 13 or higher, and in 51% of patients with an RFS score of 7 or higher. However, the sensitivity and specificity of pepsin in saliva were relatively low, at 78% and 53%, respectively. In our present study, when MMP-7 activity was used as a marker, the sensitivity and specificity were 71.43% and 79.75%, and when the two markers, MMP-7 and pepsin, were combined, the sensitivity and specificity increased to 80% and 82.28%, respectively. These results indicate that the disease predictability is better when MMP-7 is used together with pepsin as diagnostic markers. In addition, the presented criterion can be used as a cut-off value to identify the presence or absence of the disease when developed as a diagnostic device in the future.

In summary, the protein expressions of soluble e-cadherin and MMP-7, as well as the activity of MMP-7 and pepsin, were significantly increased in saliva samples taken immediately after waking up in the morning from the study group with a score of 13 or higher on the RSI. The sensitivity and specificity of MMP-7 for predicting the possibility of an RSI of 13 or higher was higher than that of pepsin. As well, when MMP-7 and pepsin were combined, this sensitivity and specificity increased. Diagnosis using the activity of MMP-7 in saliva is a non-invasive and easy technique and may be an auxiliary tool to diagnose LPRD in the future.

This study has several limitations. It is difficult to confirm the diagnostic value of MMP-7 from this study because the study was conducted on healthy volunteers, not LPRD patients. Therefore, it is thought that the diagnostic real value of MMP-7 can be proved only by conducting additional studies on symptomatic patients who are diagnosed with LPRD through various diagnostic tools including RFS (reflux finding score). Furthermore, changes in MMP-7 should be evaluated by comparing before and after LPRD drug treatment.

## Supplementary Information

Supplementary figures.
