# Supplementary material for: Usefulness of matrix metalloproteinase-7 in saliva as a diagnostic biomarker for laryngopharyngeal reflux disease
Source: Sci Rep. 2021 Aug 23;11:17071. doi: 10.1038/s41598-021-96554-7 (PMC8382706; doi:10.1038/s41598-021-96554-7)

Usefulness of Matrix Metalloproteinase-7 in saliva as a diagnostic biomarker for laryngopharyngeal reflux disease.

Nu-Ri Im[1], Byoungjae Kim[1,2], Kwang-Yoon Jung[1], Seung-Kuk Baek[1].

[1]Department of Otorhinolaryngology-Head and Neck Surgery, Korea University, College of Medicine, Seoul, South Korea;

[2]Neuroscience research institute, Korea University, College of Medicine, Seoul, Republic of Korea

Supplementary Figure 1. Soluble E-cadherin protein level, MMP-7 protein level, and MMP-7 enzyme activity within saliva samples over time. (A) Schematic diagram of the saliva sampling timeline (B) Expressions of soluble E-cadherin and MMP-7 protein according to sampling time in control and study groups. (C) MMP-7 enzyme activity according to sampling time in control and study groups. Sampling time is indicated by numbers (1 to 8).

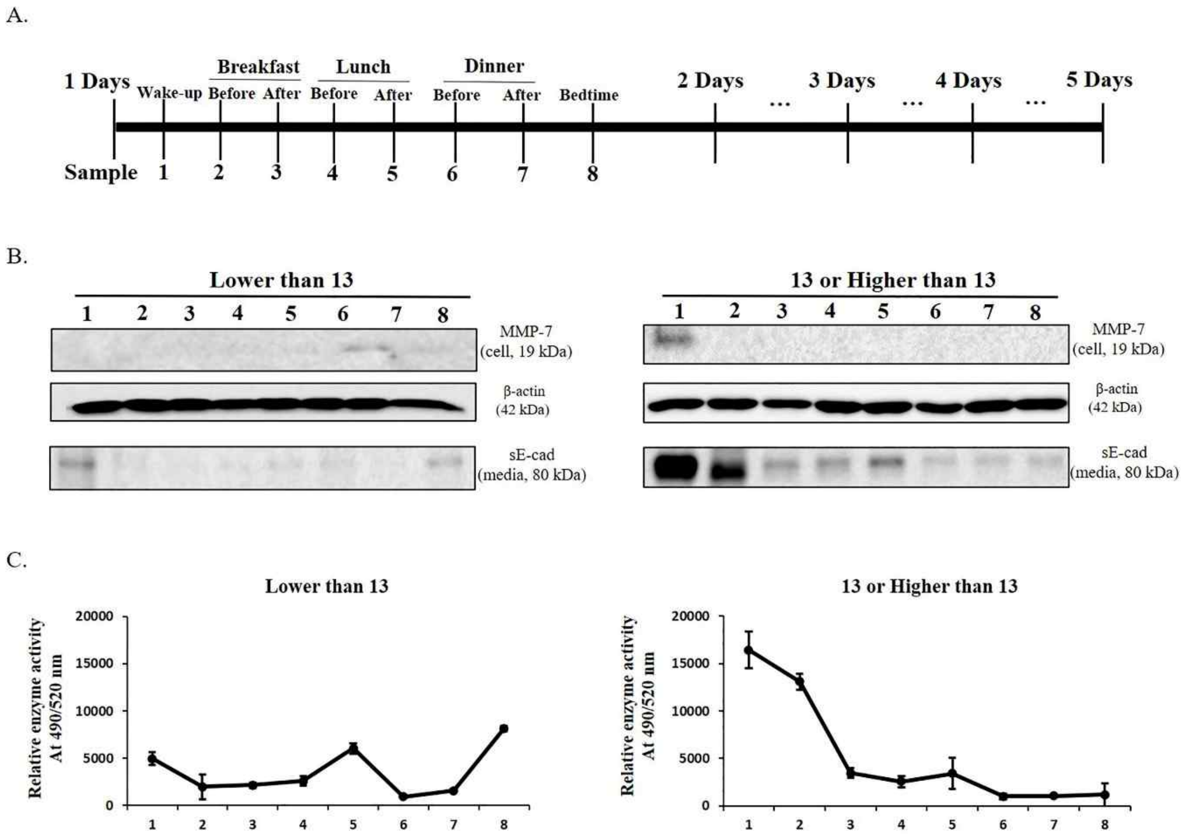

Supplementary Fig 2. Expression change of soluble E-cadherin and MMP-7 protein level, and MMP-7 enzyme activity in total saliva sample (Full-length blots/gels of Supplementary Figure 1) All blots were cropped and antibodies were attached.

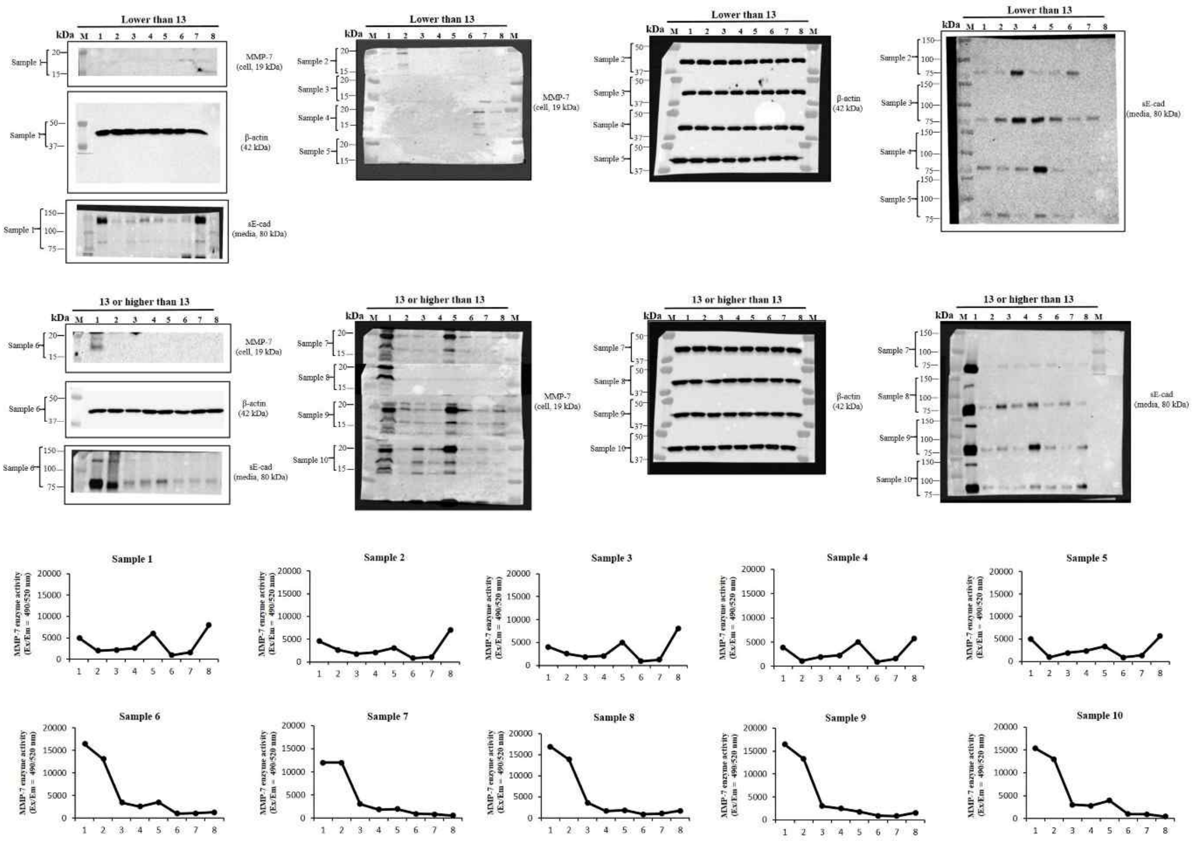

Supplement: Supplementary file 1 — Supplementary figures. [file 41598_2021_96554_MOESM1_ESM.pdf]
